# Thermal Management of SSAW Acoustofluidic Devices: Experimental and Numerical Analysis

**DOI:** 10.3390/nano15231832

**Published:** 2025-12-04

**Authors:** Andrei Megalinskii, Natasha S. Barteneva, Alexander Tikhonov

**Affiliations:** 1 Department of Physics, School of Sciences and Humanities, Nazarbayev University, Astana 010000, Kazakhstan; 2 Department of Biology, School of Sciences and Humanities, Nazarbayev University, Astana 010000, Kazakhstan; natalie.barteneva@nu.edu.kz

**Keywords:** acoustofluidics, temperature control, surface acoustic waves, acoustothermal heating, nanoparticles alignment

## Abstract

Acoustofluidic devices use Surface Acoustic Waves (SAWs) to handle small fluid volumes and manipulate nanoparticles and biological cells with high precision. However, SAWs can cause significant heat generation and temperature rises in acoustofluidic systems, posing a critical challenge for biological and other applications. In this work, we studied temperature distribution in a Standing Surface Acoustic Wave (SSAW)-based PDMS microfluidic device both experimentally and numerically. We investigated the relative contribution of Joule and acoustic dissipation heat sources. We investigated the acoustofluidic device in two heat dissipation configurations—with and without the heat sink—and demonstrated that, without the heat sink the temperatures inside the microchannel increased by 43 °C at 15 V. Adding the metallic heat sink significantly reduced the temperature rise to only 3 °C or less at lower voltages. This approach enabled the effective manipulation and alignment of nanoparticles at applied voltages up to 15 V while maintaining low temperatures, which is crucial for temperature-sensitive biological applications. Our findings provide new insights for understanding the heat generation mechanisms and temperature distribution in acoustofluidic devices and offer a straightforward strategy for the thermal management of devices.

## 1. Introduction

Microfluidic devices have become powerful tools for handling small fluid volumes in channels that range from tens to hundreds of micrometers wide [1]. They offer important benefits such as reduced reagent use, automation, and portability, which make them useful in biomedical diagnostics. A rapidly emerging subfield is acoustofluidics, where surface acoustic wave (SAW) technology has been applied to manipulate fluids and particles in the nano-micro scale. In typical acoustofluidic devices, SAWs are generated by interdigital transducers (IDTs), patterned on piezoelectric substrates such as Lithium Niobate (LiNbO_3_) [2,3,4]. SAWs create a controlled fluid motion and can impact microparticles or cells with high precision through acoustic radiation forces [5,6,7,8,9]. By depositing two IDTs facing each other, Standing Surface Acoustic Waves (SSAWs) can be generated, which are widely used for particle trapping, patterning, and sorting [10,11].

SAWs can cause significant heat generation and temperature rise in acoustofluidic systems, which may pose a critical challenge for biological applications [2,12]. Temperature is a key factor influencing physical, chemical, and biological processes in microfluidic operations. If not properly controlled, it can cause protein denaturation, cell damage, or unstable flow behavior [13,14,15] or even thermotaxis [16,17]. Experiments have shown that SAW actuation can increase the temperature of the microchannel fluid up to 80 °C [18], which exceeds the safe limit for living organisms [19,20]. Thermotaxis relates to the ability of a cell to move by sensing a temperature gradient formed by differences in temperature [21,22]. Several microfluidic devices have been utilized for cell selection based on cellular ability to swim in chemical (chemotaxis) and thermal differences (thermotaxis) [2,22,23]. Therefore, accurate temperature control is essential for the stable and biocompatible operation of acoustofluidic devices.

The primary contributors to overall heating in SAW devices are two mechanisms: acoustothermal heating in the fluid and flow channel material and Joule heating caused by electrical losses in the IDTs.

Acoustothermal heating in liquids occurs when part of the SAW leaks into the liquid and dissipates as heat due to viscous friction [24,25]. This effect is strongly dependent on the fluid’s viscosity [26]. In PDMS-based devices, acoustothermal heat is generated inside the polydimethylsiloxane (PDMS) layer because the polymer absorbs acoustic energy [27]. The amount of heating depends on the acoustic amplitude, frequency, and PDMS thickness [10,28].

Joule heating in the IDTs results from electrical dissipation into heat and contributes to SAW devices’ temperature rise. Unlike the acoustic dissipation, Joule heating is localized near the IDTs, and therefore its impact on the temperature at a specific device position strongly depends on the distance from the electrodes. Zheng et al. experimentally studied heating in droplets on a LiNbO_3_ substrate in SSAW-driven systems [25]. Wang et al. experimentally studied the relative contribution of Joule vs. acoustothermal heating for a SAW microfluidic device [13].

To reduce unwanted heating and improve biocompatibility, several temperature control methods have been tested. Active cooling with Peltier elements can help remove heat from the substrate [2,14,29,30]. Increasing flow velocity also improves convective cooling, lowering fluid temperature [18]. A simpler and highly effective approach is to place a metallic heat sink under the substrate, which efficiently removes heat and significantly reduces the device’s temperature [30,31]. It was also demonstrated that highly conductive adhesive materials between the piezoelectric substrate and the heat sink, such as silver paste, improve thermal contact, helping to further reduce substrate temperature [26].

However, to date, systematic studies of temperature distribution across acoustofluidic devices at different heat dissipation configurations are lacking. To the best of our knowledge, no temperature measurements have been performed for a SSAW device with a microchannel in PDMS. For a typical SSAW device with a microchannel inside a PDMS block, control of the microchannel temperatures at low levels suitable for biological applications is important.

In addition to experiments, numerical modeling of the temperature distribution in acoustofluidic devices provides valuable insight into heat generation mechanisms and temperature distribution, and may guide the design of SAW-based devices for improved thermal control. Most numerical studies simulate temperature fields in devices with propagating SAWs [26,31,32]. Only a few studies address SSAW-based devices. Das et al. developed a model to solve heat transfer equations for SSAW-driven acoustothermal heating of a Newtonian liquid in a microchannel [33], while the computational domain was restricted to only the fluid region. Taatizadeh et al. showed the impact of PDMS wall thickness on SSAW-driven temperature rise inside the microchannel [10].

Some studies have employed numerical modeling to study the relative contributions of different heat sources in acoustofluidic devices: Li et al. modeled droplet heating, including the effects of Joule heating and acoustic dissipation, numerically comparing these electrical and acoustic heat sources [32]. In the study by Huang et al., a numerical simulation of the real-time dynamics of temperature distribution in a droplet was shown to be in good agreement with experimental results [26].

Despite these valuable insights, combined experimental and numerical studies of temperature rise in acoustofluidic devices are lacking. Numerical results strongly depend on the device’s surrounding environment, as well as its geometrical and physical properties. Even a slight change in the parameters that regulate heat dissipation into the environment can significantly alter numerical results. Adopting some parameters of the numerical model from experimental data provides a more accurate numerical description and physical interpretation of the temperature distributions, heating mechanisms, and relative contributions of different heating sources.

In this work, we study the temperature distribution in SSAW-based LiNbO_3_-PDMS microfluidic devices both experimentally and numerically. We investigate the relative contribution of Joule and acoustic dissipation sources. We study the acoustofluidic device in two configurations—with and without a heat sink—and demonstrate that, without the heat sink, the temperatures inside the microchannel increases by 43 °C at 15 V. Adding the metallic heat sink reduces the temperature rise to only 3 °C. We also experimentally study nanoparticle manipulation and alignment driven by the acoustic field in device microchannels at different IDTs voltages. We achieve the effective alignment of nanoparticles along SSAW pressure nodes while maintaining the temperature rise in the microchannel to the low 3 °C.

Our approach provides a detailed assessment of the temperature distribution, heating mechanisms, and temperature control in the LiNbO_3_-PDMS device under SSAW, establishing practical guidelines for controlling and reducing heating effects and improving device safety and reliability in sensitive biological applications.

## 2. Materials and Methods

### 2.1. Device Fabrication

**Figure 1:**
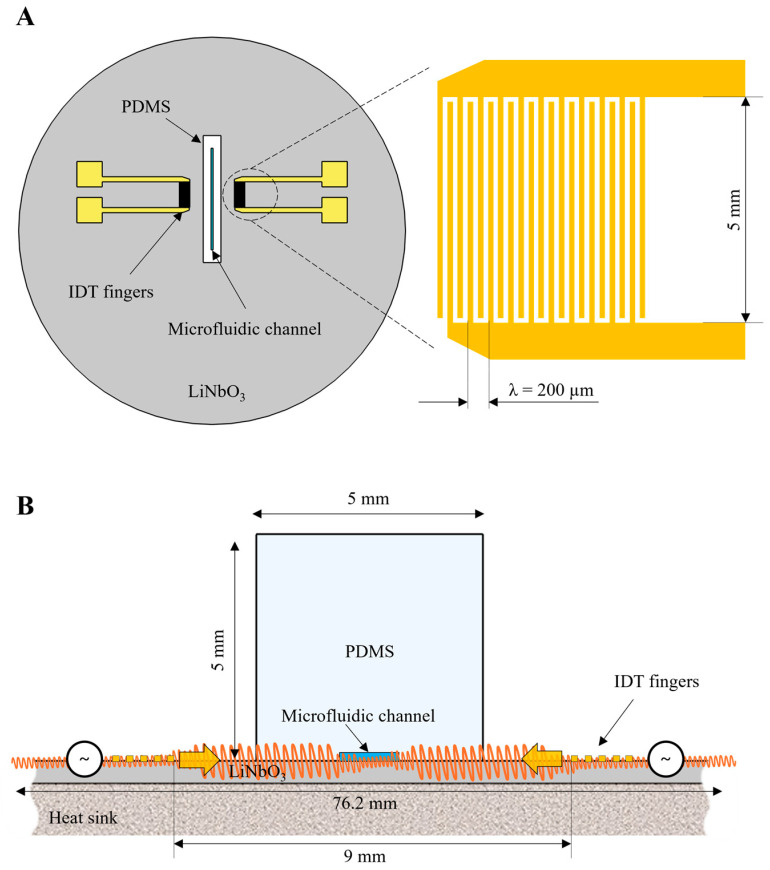
Schemes of the LiNbO_3_-PDMS acoustofluidic device used in experimental setup and numerical model. (**A**) Device top view (**left**) and zoom-in area of IDT fingers (**right**); (**B**) cross-section view of the zoomed-in part of the device. Heat sink under the LiNbO_3_ wafer is shown. In temperature measurements two device configurations will be used: with and without the heat sink.

Our acoustofluidic device consists of a single straight microfluidic channel inside a PDMS block attached to a LiNbO_3_ wafer with IDTs (Figure 1A). To generate standing acoustic waves, a pair of IDTs was positioned opposite each other at a distance of 9 mm.

IDTs were fabricated on a 3-inch 128° Y-cut LiNbO_3_ wafer using standard mask lithography methods (Karl Suss MA6, Garching, Germany), with 21 fingers per IDT and a finger length of 5 mm. The distance between separate IDT fingers is 50 µm (corresponding to a wavelength of 200 µm), with a tolerance of 0.5 µm. A LiNbO_3_ wafer with 2 µm thick photoresist layer (ma-N 1420) was exposed at 405 nm with a dose of 550 mJ/cm^2^, followed by a developing for 60 seconds (ma-D 533S). Then, the electron-beam evaporation method (Kurt J. Lesker PVD200, Jefferson Hills, PA, USA) was used to deposit a 5 nm titanium adhesion layer followed by a 50 nm gold layer. Lift-off was performed in acetone using an ultrasonic bath.

A simple single straight microfluidic channel 2 cm long, with a cross-section of 50 µm in height and 800 µm in width in PDMS (Figure 1B), was fabricated using standard soft-photolithography methods. First, microfluidic channel molds were fabricated on a silicon wafer using standard SU-8 photolithography. A 4-inch silicon wafer with 50 µm thick photoresist layer (SU-8 2050) was pre-baked at 100 °C for 8 min, then exposed at 405 nm with a dose of 175 mJ/cm^2^, followed by post-baking at 100 °C for 10 min and developing for 6 minutes. Then the patterned mold was cast in PDMS (Sylgard 184) and baked at 60 °C for 2 h. A polymerized PDMS block with a cross-section of 5 × 5 mm was cut out of the casted mold, and inlet and outlet holes were then punched at both ends to allow tubing connection.

To attach the LiNbO_3_ wafer with patterned IDTs to the PDMS block, they were first both treated with oxygen plasma at a flow rate of 400 sccm for 5 min at 30 W power. After plasma activation, the LiNbO_3_ and PDMS were aligned and attached so that the microchannel was positioned between the IDTs.

### 2.2. Experimental Setup

The temperature experiments were performed in two configurations, one using (a) a bare LiNbO_3_ wafer and another using (b) an assembled LiNbO_3_-PDMS acoustofluidic device, where a PDMS block with a microfluidic channel was attached on top of the LiNbO_3_ wafer.

**Figure 2:**
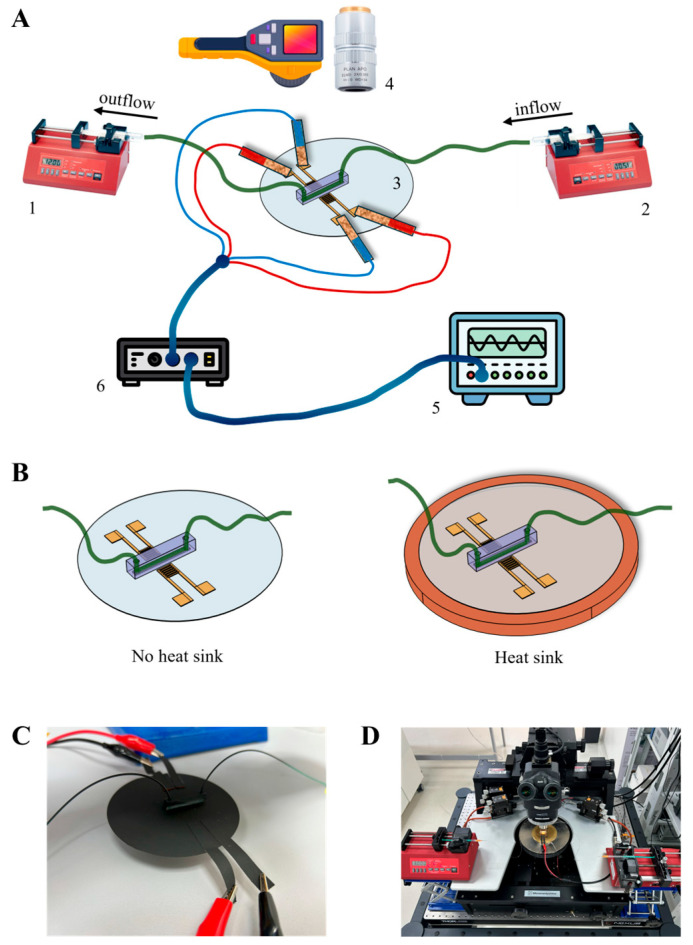
Experimental setups. (**A**) Schematic of the experiment to measure temperature distribution: 1, 2—syringe pumps for controlling inflow and outflow through the PDMS microchannel; 3—assembled LiNbO_3_-PDMS acoustofluidic device; 4—IR camera and long WD objective for particle’s distribution detection; 5—RF generator; 6—RF amplifier (×50); (**B**) assembled LiNbO_3_-PDMS acoustofluidic device suspended in air (no heat sink), and with metal plate under the LiNbO_3_ wafer (heat sink); (**C**) photo of covered with mate black paint LiNbO_3_-PDMS acoustofluidic device for temperature measurements; (**D**) photo of the reflective microscopy setup to study SSAW-driven particle alignment. Here, the assembled LiNbO_3_-PDMS acoustofluidic device is on the heat sink.

Both configurations were driven by an AC sinusoidal signal generator (Keysight 33600A, Santa Rosa, CA, USA) connected to an RF amplifier with ×50 gain (Electronics & Innovation 403LA, Rochester, NY, USA). Copper tape was used to attach the wires to the IDT contact pads. Alternating voltages ranging from 1 to 15 V were applied at the IDT resonance frequency (19.73 MHz for bare LiNbO_3_ wafer and 19.28 MHz for assembled LiNbO_3_-PDMS acoustofluidic device). For the assembled LiNbO_3_-PDMS acoustofluidic device, the flow through the microchannel was controlled by syringe pumps (New Era model 1000, Suffolk County, NY, USA) at a rate of 3 µL/min (Figure 2A).

Temperature measurements were conducted under two thermal dissipation conditions: (a) “no heat sink”—the device was suspended in air (Figure 2B, left), (b) “heat sink”—the device was placed on a (brass thermal chuck) plate (D = 101.1 mm, h = 3.9 mm) serving as the heat sink (Figure 2B, right). It should be noted that a heat sink could be any solid material with high heat conductivity and capacity in direct contact with the backside of the wafer.

For temperature characterization, a thermal camera (Fluke Ti480 PRO, Everett, WA, USA) was positioned above the system. In these experiments, the device’s surface was coated with black mate paint (Figure 2C) to minimize emissivity artifacts. To obtain accurate temperature measurements across the device, it is essential to coat it with black paint, especially since it includes highly transparent components such as LiNbO_3_ and PDMS.

Experiments on SSAW-driven nanoparticle alignment in the microchannel were performed using polyethylene nanospheres (Cospheric, 200 nm–9900 nm, 0.95 g/cc). For microscopic images of particle distribution in the device’s microchannel, a probe station microscope (Motic PSM 1000, Kowloon, Hong Kong) was used, with an extra-long working distance objective (2X/0.055, WD = 34 mm). Since the bottom side of the device’s LiNbO_3_ wafer was placed on a non-transparent high heat conductivity plate, traditional transmission microscopy could not be used and instead we utilized reflected light microscopy (see the photo of the experimental setup, Figure 2D).

### 2.3. Numerical Simulations

Acoustic pressure and temperature distribution in the bare LiNbO_3_ wafer and in the LiNbO_3_-PDMS acoustofluidic device was calculated by a stationary 2D numerical model developed in COMSOL Multiphysics software (v6.2), using the finite element method. The geometric dimensions were the same as in the experimental setup (see Figure 1B).

The COMSOL modules used in simulations were as follows: Solid Mechanics, Electrostatics, Pressure Acoustics (for the LiNbO_3_-PDMS acoustofluidic device configuration), and Heat Transfer. The main material properties for the LiNbO_3_, PDMS, and water (in the microfluidic channel) used in the simulations are shown in Table 1.

For LiNbO_3_ elasticity and coupling matrices, we used the following parameters (Table 2 and Table 3), taken from Pyroelectric Detector 2D axisymmetric model in Comsol Blog (Application ID: 110101):

The relative permittivity coefficients are $\varepsilon_{rS11}=43.6$, $\varepsilon_{rS22}=43.6$, $\varepsilon_{rS33}=29.16$, and $\varepsilon_{rSij}$ = 0. Total pyroelectric coefficients are $p_{ET1}=0$, $p_{ET1}=0$, and $p_{ET3}=-8.3\times 10^{-5}$ C/(m^2^·K).

To model the surface acoustic waves propagation in the LiNbO_3_ crystal in the chosen 2D cross-section (Figure 1B), a Euler angles rotation system was applied to the Solid Mechanics module, with the β angle rotated to −128°. A mechanical impedance (1) was applied to the side and bottom surfaces of the LiNbO_3_ crystal domain:

(1) $$d_{im}=\frac{\rho (c_{p}+c_{s})}{2}$$

where ρ is the density of the material, $c_{p}$ is equivalent speed of pressure wave, and $c_{s}$ is equivalent speed of shear wave.

The attenuation coefficients for PDMS and liquid were taken from [34]. Reference impedance for Electrostatics module was set to 50 Ohm. In the Heat Transfer module, the surface emissivity was set to 0.95 for both LiNbO_3_ and PDMS surfaces. Maximum mesh size element was chosen as 20 µm for all domains, which is 1/10 of the surface wavelength.

## 3. Results

### 3.1. Experimental Temperature Measurements in Bare LiNbO_3_ Wafer with IDT and LiNbO_3_-PDMS Acoustofluidic Device

We measured temperature distribution maps in two experimental setups: (1) a bare LiNbO_3_ wafer with IDTs and (2) an assembled PDMS block (with a microchannel) attached to a LiNbO_3_ wafer with IDTs. In both setups, temperature measurements were performed with and without the heat sink. Temperature maps were recorded after turning on AC current for five minutes, so the temperatures stabilized to the equilibrium values. Infrared thermal maps on the surface were recorded at the following applied voltages: 1 V, 2 V, 5 V, 10 V, and 15 V.

**Figure 3:**
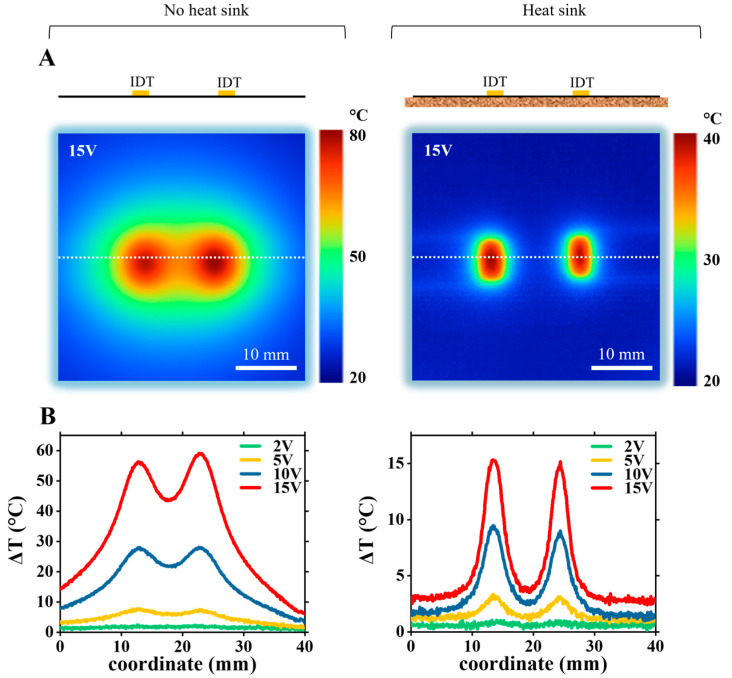
Experimental measurements of temperature distribution maps on bare LiNbO_3_ wafer with IDTs. (**A**) Thermal maps of temperature distribution (absolute values) at 15 V applied to IDTs. **Left**: no heat sink; **right**: with the heat sink. (**B**) Temperature rise profiles taken along the dashed white lines (shown in (**A**)) for the range of applied voltages, 2–15 V.

For the bare LiNbO_3_ wafer with IDTs, the thermal maps at 15 V applied voltage are shown at Figure 3A, left—without the heat sink under the wafer, right—with the heat sink. Two bright red spots in the center of each image indicate the highest temperatures and correspond to the IDT region. Without the heat sink, the maximum temperature increased to ~80 °C, while with the heat sink, the temperature rose to a much lesser value of ~35 °C. These temperature hot spots most likely occur due to Joule heating at IDTs [13].

**Figure 4:**
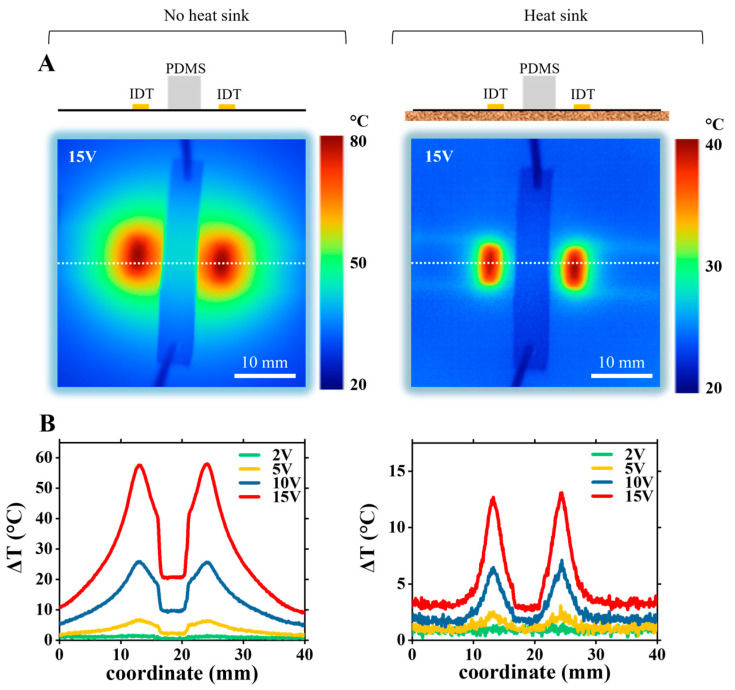
Experimental measurements of temperature distribution maps on assembled LiNbO_3_-PDMS device. (**A**) Thermal maps of temperature distribution (absolute values) at 15 V applied to IDTs. **Left**: no heat sink; **right**: with the heat sink. (**B**) Temperature rise profiles taken along the dashed white lines (shown in (**A**)) for the range of applied voltages, 2–15 V.

In the area between the IDTs, the temperature increased to ~65 °C without the heat sink, while with the heat sink, it is less and around 25 °C. Figure 3B shows the temperature rise distribution profile along the device’s surface (along the dashed white line on Figure 3A) at different applied voltage values: 2 V, 5 V, 10 V, and 15 V. The left figure shows temperature rise profiles without the heat sink, the right figure—with the heat sink. To obtain the temperature rise profile values, the temperature profile data at ambient room temperature (no voltage applied to the IDTs) was subtracted from each temperature profile. The results of temperature measurement at different applied voltages for the assembled LiNbO_3_-PDMS device are shown in Figure 4. Temperature distribution maps of the device at applied 15 V are shown in Figure 4A, without the heat sink (left figure), and with the heat sink (right figure). Figure 4B shows the temperature rise profiles along the line across the device, shown by the white dashed line in Figure 4A.

For the assembled device, the temperatures of the device areas away from the PDMS block are similar in value to the bare LiNbO_3_ wafer with IDTs. This indicates that attaching the PDMS block does not significantly affect the heating and temperature distribution across the wafer. The temperatures measured from the top of the PDMS block are naturally lower, as PDMS is a relatively weak thermal conductor. At 15 V voltage applied to IDTs, without the heat sink, the temperature rises at the PDMS top surface by ~22 °C, while with the heat sink, the temperature rises by a much smaller value of ~3 °C.

Our results indicate that the largest temperature rise occurs near the IDTs, indicating that Joule heating is the dominant source of heating. Nevertheless, the relative role of Joule vs. acoustothermal heating at different areas of the device remains unclear. Also, the temperatures inside the PDMS block at the microfluidic channel cannot be measured in the current experimental setup and remain unknown. To find these temperatures at the microchannel, we created a numerical simulation model, calibrated it with our experimental results, and calculated the temperature distributions across the device, including the microfluidic channel area.

### 3.2. Numerical Simulation of Acoustic Pressure Distribution

To calculate the distribution of temperature in the acoustofluidic device, we took into account two different sources of heat: acoustic dissipation of SSAW (acoustothermal heating) and Joule heat at IDTs. To find the acoustothermal heating in the device, the first step is to calculate the acoustic pressure distribution across the device.

The acoustic pressure distribution was numerically simulated in both a bare LiNbO_3_ wafer with IDTs and an assembled LiNbO_3_-PDMS acoustofluidic device. In the computational model, IDTs were presented as line segments on the surface of the LiNbO_3_ wafer, with 1–15 AC voltage and 19.33 MHz frequency applied.

**Figure 5:**
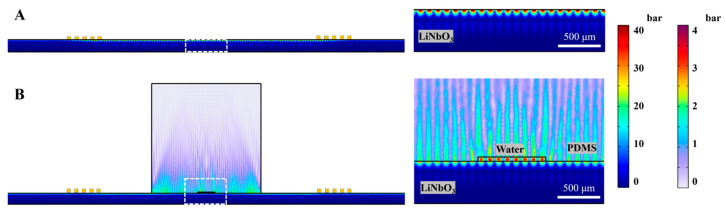
Numerical simulation of pressure distribution in (**A**) bare LiNbO_3_ with IDTs and (**B**) LiNbO_3_-PDMS device (absolute values, 15 V applied). Dashed white rectangles (left) represent the zoomed-in area on the right side.

Figure 5A shows the pressure map of the bare LiNbO_3_ wafer for 15 V applied to IDTs. The zoomed-in part between IDTs is shown on the right. For the LiNbO_3_-PDMS acoustofluidic device (Figure 5B), the pressure map also demonstrates the penetration of leaky waves from the LiNbO_3_ substrate into the PDMS block and flow channel. To ensure that the model captures the relevant processes, the pressure node distribution map and absolute pressure values in PDMS were qualitatively compared to previously published works [6,34], showing similar spatial patterns and magnitudes of acoustic pressure. It can be seen that pressure in the microfluidic channel is higher than in the surrounding PDMS due to its lower attenuation coefficient [34]. For simplification, the shear rate of flow in the microfluidic channel was neglected.

It should be noted that surface pressure wave parameters (such as amplitude, phase, and attenuation) are highly sensitive to the initial and boundary conditions of the model, as well as mesh density. In real devices, the reflectance from the wafer’s boundaries, losses of AC signal in cables, and limitations in accuracy during the fabrication process have a significant impact on the pressure node distribution in the wafer.

However, some fundamental principles remain valid. For example, SSAWs have a higher amplitude between the IDTs; the wavelength and speed of the propagating waves are defined by the geometry of the IDTs and the material properties.

### 3.3. Numerical Simulation of Temperature Distributions

The heat in the acoustofluidic device was assumed to come from two different sources: acoustic dissipation of SSAW (acoustothermal heating) and Joule heat at IDTs. The heat sources resulting from acoustic wave dissipation in the LiNbO_3_ wafer (2) and PDMS block (3) were defined by the expressions adapted from [35]:

(2) $$Q_{{LiNbO}_{3}}=\frac{p_{{LiNbO}_{3}}^{2}}{2\cdot \rho_{{LiNbO}_{3}}\cdot \upsilon_{{LiNbO}_{3}}\cdot l_{IDT}}$$

(3) $$Q_{PDMS}=\frac{p_{PDMS}^{2}}{2\cdot \rho_{PDMS}\cdot \upsilon_{PDMS}\cdot l_{PDMS}}$$

where *p* is the local pressure, ρ is the density of the material, *υ* is the speed of sound, and *l* is the length of the IDT fingers and PDMS, respectively. The Joule heat at IDTs was defined as a constant heat flux originated at the IDT fingers. Volumetric acoustothermal heating of the liquid in the microfluidic channel was neglected due to its relatively small volume compared to that of the LiNbO_3_ and PDMS.

**Figure 6:**
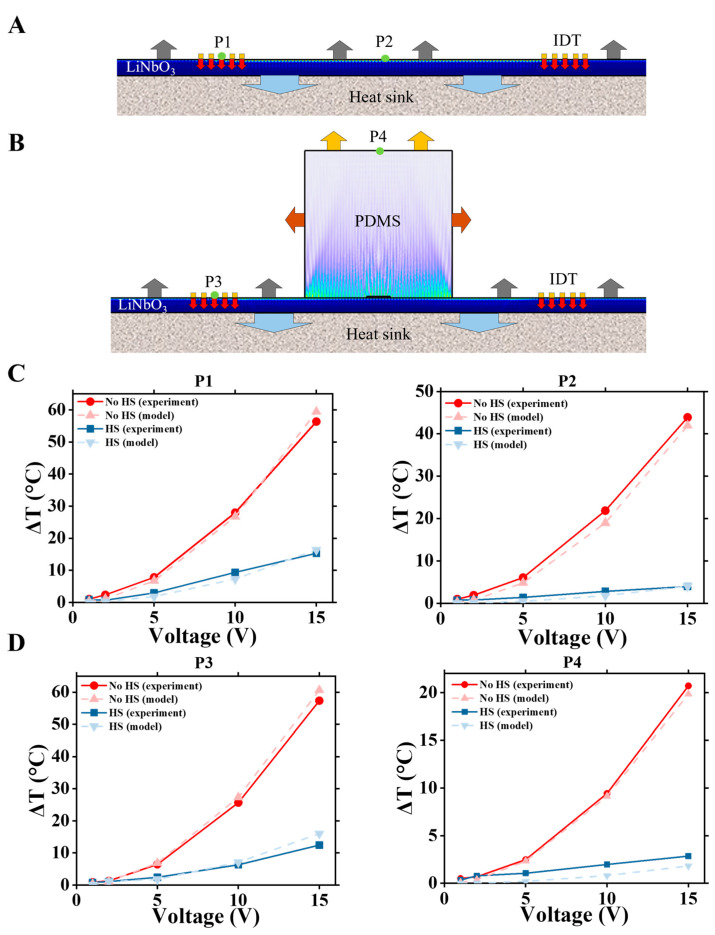
Experimental and numerical temperature rises in two points, P1 and P2, of bare LiNbO_3_ with IDTs, and two points, P3 and P4, of LiNbO_3_-PDMS device. (**A**) Heat fluxes across the boundaries are shown with arrows in the bare LiNbO_3_ wafer with IDTs. Here, gray arrows indicate heat removal from the wafer’s top surface; blue arrows indicate heat removal from the bottom surface; red arrows indicate the Joule heat flux from the IDTs. (**B**) Heat fluxes in the LiNbO_3_-PDMS acoustofluidic device. Here, in addition to gray and blue arrows, brown arrows indicate heat removal from the side surfaces of PDMS; yellow arrows indicate heat removal from the top surface of PDMS. (**C**,**D**) Comparison of experimental and modeled temperature rise at points P1-P4, shown for configurations without the heat sink (red lines) and with the heat sink (blue lines).

To simulate the temperature distribution in both the bare LiNbO_3_ wafer with IDTs and the LiNbO_3_-PDMS acoustofluidic device, heat dissipation fluxes from and across the surfaces of the LiNbO_3_ wafer and PDMS block needed to be properly specified. Heat dissipation flux values across the LiNbO_3_ and PDMS surfaces strongly depend on specific experimental conditions. For example, air convection near the surfaces may significantly vary, strongly affecting heat dissipation into the air environment. To capture the balance between heat generation and heat removal to the surroundings, three types of heat dissipation were considered: heat removal from the LiNbO_3_ surface (Figure 6A), heat removal from the vertical walls of the PDMS block (Figure 6B), and from the horizontal PDMS top surface. The typical range of these values was estimated from [36]. To simulate the presence of the heat sink, an increased heat dissipation flux was assigned to the bottom surface of the LiNbO_3_ wafer.

We fitted (calibrated) our numerical model to experimental data using a parametrical sweep analysis. The numerical values used for calibration are summarized in Table 4. To choose the values for these parameters of our numerical model, we varied them to find the best fit to experimentally measured temperatures at four points, indicated in Figure 6A,B. Two points, P1 and P2, are located at the center of the IDT fingers region and in the middle between IDTs for the bare LiNbO_3_ wafer with IDTs (Figure 6A). For the assembled LiNbO_3_-PDMS device, point P3 is located at the center of the IDT fingers region and point P4 is in the middle of the PDMS top surface (Figure 6B). The results of the temperature simulations using our calibrated numerical model are plotted, together with experimental data in Figure 6C,D, both for the configurations without a heat sink (no HS) and with the heat sink (HS).

Please note that the experimental data at points P1, P2, P3, and P4, presented in Figure 6 and used for numerical model calibration, is shown in Figure 3 and Figure 4 and discussed in Section 3.1. In Figure 3 and Figure 4, we plot full maps of experimental data across the device, where data at points P1 and P3 represent measurements at the center of the IDTs and data at points P2 and P4 correspond to measurements taken midway between the IDTs.

### 3.4. Numerical Simulations of the Temperature Distribution Inside the Devices: Relative Contributions of Joule vs. Acoustic Dissipation Heat

**Figure 7:**
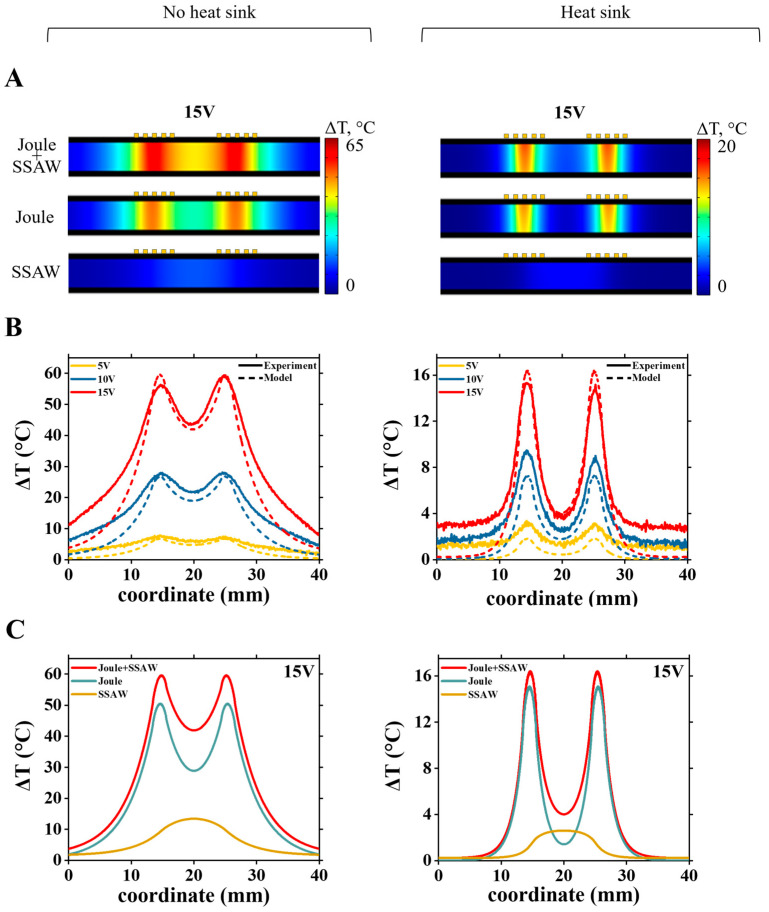
Analysis of the temperature profile across the surface of the bare LiNbO_3_ wafer with IDTs. (**A**) Simulated temperature maps at 15 V showing the effect of the heat sink on heat distribution near the IDTs, for different heat sources (images are stretched along the vertical direction, wafer thickness is 500 μm); (**B**) Comparison of experimental (solid lines) and simulated (dashed lines) temperature distributions along the LiNbO_3_ surface without and with the heat sink, for 5 V, 10 V and 15 V applied to IDTs; (**C**) Simulated temperature profiles at 15 V without and with the heat sink. Red, blue and yellow curves are, correspondingly, results for Joule and acoustothermal heat (Joule + SSAW) source together, only Joule heat source and only acoustothermal heat (SSAW) source.

The calibrated numerical model provides an accurate reconstruction of the temperature distribution data inside and across the devices. Figure 7A shows the calculated temperature maps at 15 V across bare LiNbO_3_ wafer with IDTs, without (left figure) and with (right figure) the heat sink. Figure 7B shows both the calculated and experimentally measured surface temperature profile (along the white dotted line at Figure 3A) at different applied voltages. The results show that using the heat sink effectively reduces the temperature across the device.

With the numerical model, we can study the relative contribution of Joule vs. acoustothermal heat sources. Simulations of temperature rise across the bare LiNbO_3_ wafer with IDTs at applied 15 V are shown in Figure 7C. Without a heat sink, the Joule heating dominates across the device (Figure 7C, left). Although Joule heating is localized at the IDT region, it spreads out across the LiNbO_3_ wafer through thermal conductivity and becomes dominant even in the areas between the IDTs. Interestingly, adding the heat sink effectively dissipates thermal energy across the device, and although Joule heating still dominates in the IDT region, in the region between IDTs, acoustothermal heating prevails.

**Figure 8:**
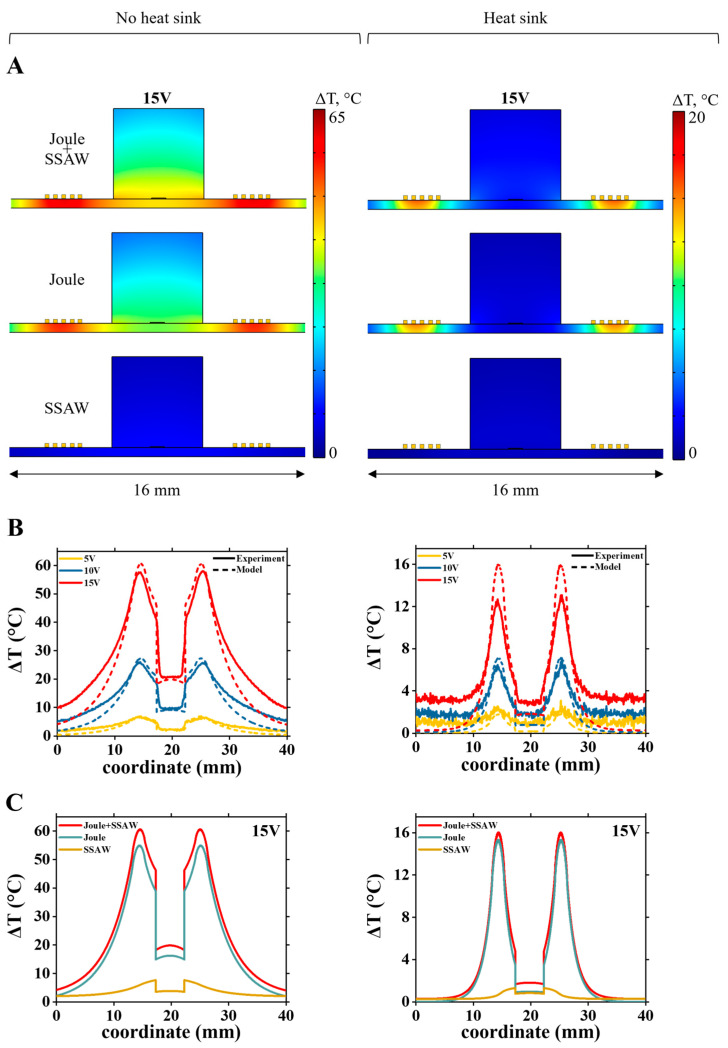
Analysis of heat distribution in the LiNbO_3_-PDMS device. (**A**) Simulated temperature maps at 15 V showing the effect of the heat sink on heat distribution near the IDTs for different heat sources. (**B**) Comparison of experimental (solid lines) and simulated (dashed lines) temperature distributions along the LiNbO_3_-PDMS surface without and with the heat sink, for 5 V, 10 V, and 15 V applied to IDTs. (**C**) Simulated temperature profiles at 15 V without and with the heat sink. Red, blue, and yellow curves are, correspondingly, results for Joule and acoustothermal heat (Joule + SSAW) source together, only Joule heat source, and only acoustothermal heat (SSAW) source.

Similarly, Figure 8A shows calculated temperature distribution maps at 15 V for the LiNbO_3_-PDMS device without (left) and with the heat sink (right). Figure 8B shows both calculated and experimentally measured surface temperature profile (along the white dotted line at Figure 4A) at different applied voltages. Comparing relative Joule and acoustothermal heat source contributions in the LiNbO_3_-PDMS device without the heat sink, almost all temperature rise across the device comes from Joule heat (Figure 8C, left). With the heat sink, the Joule heat dominates everywhere except for the top surface of the PDMS block, where Joule heat and acoustothermal heat sources contributions are almost equal (Figure 8C, right).

**Figure 9:**
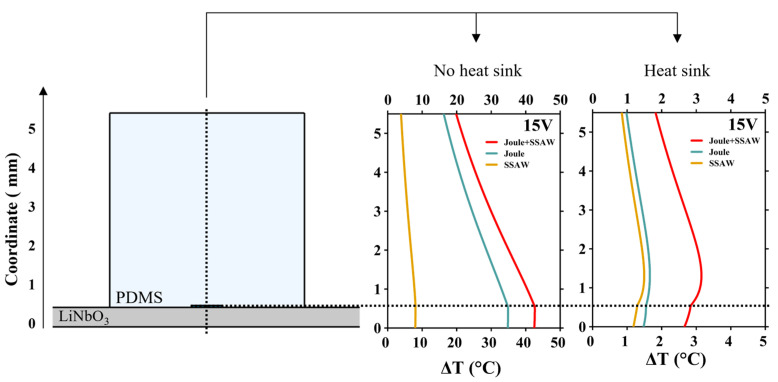
Simulated temperature distribution profiles in the LiNbO_3_-PDMS device at 15 V, with and without the heat sink. Vertical cross-section temperature profiles (black dashed line on the right scheme) illustrating temperature values through the substrate and PDMS chamber with microfluidic channel.

To study the relative contribution of Joule vs. acoustothermal heat inside the PDMS block and at the microchannel, we calculated temperature vertical profiles inside the LiNbO_3_-PDMS device at an applied 15 V, without and with the heat sink (Figure 9). Temperature rise was calculated along the cross-section profile through the center of the device, indicated by the vertical black dashed line on the right scheme Figure 9. The horizontal dashed line indicates the position of the microchannel. Our results demonstrate that the temperature rise (at 15 V) within the microfluidic channel remains at ~45 °C without the heat sink and decreases to ~3 °C when the heat sink is applied. Interestingly, without the heat sink, Joule heating greatly dominates at the microchannel, while after adding the heat sink, the relative contribution of acoustothermal heating strongly increases and becomes comparable to the Joule heating contribution.

### 3.5. Alignment of Nanoparticles in Acoustofluidic Device

**Figure 10:**
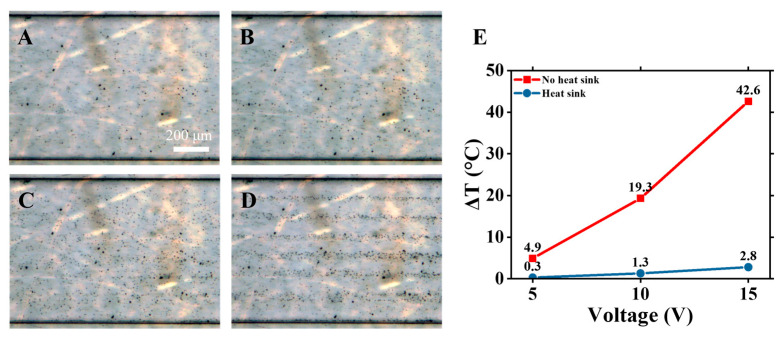
Distribution of polystyrene nanoparticles of different sizes (200 nm–9.9 μm range of diameters) in the microfluidic channel at different voltages applied to IDTs: (**A**) 0 V, (**B**) 5 V, (**C**) 10 V, (**D**) 15 V. Flow rate: 3 μL/min. (**E**) Numerically calculated temperature rise inside the microfluidic channel for different applied voltages (5, 10, 15 V) without the heat sink (red line) and with the heat sink (blue line).

In typical experiments of microparticle sorting using acoustofluidic devices, particle alignment in the microchannel with an acoustic field improves with the increase in applied AC voltage (see, for example, Taatizadeh et al. [10]). At the same time, with the AC voltage applied to IDTs, the temperatures in the device and inside the microchannel rise. To show the degree of particle alignment at different voltages, a series of experiments with polystyrene beads were conducted. We pumped a solution of microspheres with diameters ranging from 200 nm to 9.9 μm through the microfluidic channel and recorded reflected microscopy images of particles distribution at three different applied voltages: 5, 10, and 15 V. Clear particle alignment along the pressure nodes appears only when high (15 V) voltage is applied, while at 10 V, particle alignment is noticeable, but much weaker (Figure 10A–D).

In many applications, especially biological applications, it is important to maintain a low temperature rise inside the microchannel (sometimes no more than 1–3 °C) while achieving effective acoustically driven particle sorting and alignment. As we demonstrated above, the temperature rises in our device’s microchannel by ~3 °C when the 15 V voltages are applied to the device with the heat sink, while at 5 V and 10 V, the temperature increases by only 0.3° and 1.3°, respectively (Figure 10E). For our device, 10 V and 15 V voltages are above the threshold to achieve effective microparticle and cell sorting while maintaining low temperatures inside the microfluidic channel, which is suitable for many applications.

## 4. Discussion and Conclusions

In conclusion, we studied temperature distribution and temperature control in SSAW-based LiNbO_3_-PDMS microfluidic devices both experimentally and numerically. We studied temperatures of the SSAW acoustofluidic device at a range of applied AC voltages to IDTs, and utilized two configurations: without the heat sink (LiNbO_3_ wafer device suspended in air) and with the heat sink under the wafer. Our experiments demonstrated a significant increase in temperatures in the microfluidic channel in the device without the heat sink (up to 45 °C at 15 V voltages applied to IDTs), consistent with prior reports for SAW devices [15]. Integration of the metallic heat sink reduced the temperature elevation to ~3 °C in the channel.

Simulations using our calibrated numerical model show that Joule heating originating at the IDTs area is the dominant heating mechanism for the device without the heat sink. With the heat sink, the relative importance of acoustothermal heating due to acoustic energy dissipation becomes more pronounced and becomes comparable to or larger than Joule heating in the area between the IDTs. More generally, our numerical simulation model provides a quantitative estimation of the relative contributions of Joule and acoustothermal heating across the device, which may help in designing future devices with effective temperature controls.

However, the physical mechanisms underlying the relative contribution shift between Joule heating and acoustothermal heating after adding the heat sink require further investigation. Joule heating is generated at the IDT area and then thermally conducted to the other device areas. Therefore, the contribution of Joule heating to the temperature rise at a specific location decreases with increasing distance between this location and the IDTs. In contrast, acoustothermal heat is generated across the whole device according to the acoustic pressure distribution, dominating on the LiNbO_3_ wafer top surface and the bottom part of the PDMS layer (Figure 5). Also, LiNbO_3_ has a higher thermal conductivity than PDMS, while the LiNbO_3_ wafer is much thinner than the PDMS layer. With the heat sink, Joule heat is efficiently drained through the wafer into the heat sink, contributing less to the device areas near the microchannel, which are away from the IDTs.

Thus, we show that simple thermal management (adding a heat sink) can suppress overall heating to a low temperature rise. This insight is crucial for any biological applications, where heating is a limiting factor for viability and cell motility. For example, for ultrasound sperm activation, maintaining temperature control is essential [37]. Sperm thermotaxis is species-specific, with a temperature range of 36–37.5 °C along the separation channel in some species [38]. These results demonstrate that acoustofluidic devices can be operated safely for biological samples if proper thermal management is implemented. Our approach complements other strategies such as external cooling or substrate modifications, but remains simple, effective, and compatible with standard LiNbO_3_ wafers.

We performed experiments on the alignment of nanoparticles in the microfluidic channel, using a device with the heat sink for temperature control. We found that using 10–15 V applied voltage enables effective alignment of nanoparticles with the acoustic field, while maintaining the temperature rise in the microchannel to a low level of 1–3 °C. In our nanoparticle alignment experiments, we used a custom-built brightfield microscopy system designed for materials science applications, operating in reflected light and equipped with long-working-distance (>3 cm) objectives. We note that, when working with biological samples and controlling the temperatures of the acoustofluidic device by placing the non-transparent heat sink under the device, transmitted light microscopy cannot be used. Moreover, conventional short-working-distance objectives often lack sufficient working distance and focal depth to focus on the microchannel positioned below the PDMS layer.

In the present work, we report the results obtained using a representative device with a specific design and fabrication recipe. A further systematic parametric study of variations in PDMS block geometry, flow rates, mechanical coupling between the LiNbO_3_ wafer and PDMS, and the efficiency of thermal contact should expand upon our current findings. We believe that our numerical model is capable of capturing these case-specific variations and predicting the corresponding thermal response. Gathering a larger set of experiments and conducting a systematic comparison can improve the robustness of the model.

Achieving precise temperature control at the microscale involves several challenges due to thermal gradients and the necessity for rapid and localized thermal adjustments. Additionally, the thermal conductivity of the substrate plays an important role in regulating heat transfer. Addressing these challenges requires optimized materials and refined device designs [23]. Our findings provide new insights into understanding the heat generation mechanisms and temperature distribution in acoustofluidic devices, and offer a straightforward strategy for device thermal management. Our approach enables the more controlled use of acoustic microfluidic devices for temperature-sensitive biological applications.

## Figures and Tables

**Table 1:** Material properties used in numerical simulations.

Model Parameters	LiNbO_3_	PDMS	Water
Heat capacity at constant pressure [J/(kg·K)]	628	1460	4188.3
Density [kg/m^3^]	4700	970	976.26
Thermal conductivity [W/(m·K)]	4.2	0.16	0.66146
Speed of sound [m/s]	3992	1030	1555

**Table 2:** Elasticity matrix, Voigt notation (Pa).

	$c_{Ei1}$	$c_{Ei2}$	$c_{Ei3}$	$c_{Ei4}$	$c_{Ei5}$	$c_{Ei6}$
$c_{E1j}$	2.02897 × 10^11^	5.29177 × 10^10^	7.49098 × 10^10^	8.99874 × 10^9^	0	0
$c_{E2j}$	5.29177 × 10^10^	2.02897 × 10^11^	7.49098 × 10^10^	−8.99874 × 10^9^	0	0
$c_{E3j}$	7.49098 × 10^10^	7.49098 × 10^10^	2.43075 × 10^11^	0	0	0
$c_{E4j}$	8.99874 × 10^9^	−8.99874 × 10^9^	0	5.99034 × 10^10^	0	0
$c_{E5j}$	0	0	0	0	5.99018 × 10^10^	8.98526 × 10^9^
$c_{E6j}$	0	0	0	0	8.98526 × 10^9^	7.48772 × 10^10^

**Table 3:** Coupling matrix, Voigt notation (C/m^2^).

	$e_{ESi1}$	$e_{ESi2}$	$e_{ESi3}$	$e_{ESi4}$	$e_{ESi5}$	$e_{ESi6}$
$e_{ES1j}$	0	0	0	0	3.69594	−2.53384
$e_{ES2j}$	−2.53764	2.53764	0	3.69548	0	0
$e_{ES3j}$	0.193644	0.193644	1.30863	0	0	0

**Table 4:** Model parameters used for calibration.

Model Parameters	No Heat Sink	Heat Sink
Heat flux from LiNbO_3_, bottom surface, W/(m^2^·K)	50	750
Joule heat from IDT at 15 V, W/m^2^	4.5 × 10^4^	
Heat flux from LiNbO_3_, top surface, W/(m^2^·K)	50	
Heat flux from PDMS, top surface, W/(m^2^·K)	20	
Heat flux from PDMS, side surfaces, W/(m^2^·K)	5	

## Data Availability

The original contributions presented in this study are included in the article. Further inquiries can be directed to the corresponding authors.

## Abbreviations

The following abbreviations are used in this manuscript:

SAW	Surface Acoustic Wave
IDT	Interdigital Transducer
LiNbO_3_	Lithium Niobate
SSAW	Standing Surface Acoustic Wave
PDMS	Polydimethylsiloxane

## References

[1] Whitesides G.M. (2006). The Origins and the Future of Microfluidics. Nature.

[2] Dos-Reis-Delgado A.A., Carmona-Dominguez A., Sosa-Avalos G., Jimenez-Saaib I.H., Villegas-Cantu K.E., Gallo-Villanueva R.C., Perez-Gonzalez V.H. (2023). Recent Advances and Challenges in Temperature Monitoring and Control in Microfluidic Devices. Electrophoresis.

[3] Go D.B., Atashbar M.Z., Ramshani Z., Chang H.-C. (2017). Surface Acoustic Wave Devices for Chemical Sensing and Microfluidics: A Review and Perspective. Anal. Methods.

[4] Peng L., Zhou Y., Guan W., Zhao F. (2025). Surface Acoustic Wave Manipulation of Fluids and Suspended Particles in Microchannels and Sessile Droplet: A Review. Capillarity.

[5] Qi M., Dang D., Yang X., Wang J., Zhang H., Liang W. (2023). Surface Acoustic Wave Manipulation of Bioparticles. Soft Matter.

[6] Mezzanzanica G., Agazzi L., Siena M., Français O., Mariani S. (2022). A Microfluidic Device Based on Standing Surface Acoustic Waves for Sorting and Trapping Microparticles. Eng. Proc..

[7] Nam J., Lim H., Shin S. (2011). Manipulation of Microparticles Using Surface Acoustic Wave in Microfluidic Systems: A Brief Review. Korea-Australia Rheol. J..

[8] Ni Z., Yin C., Xu G., Xie L., Huang J., Liu S., Tu J., Guo X., Zhang D. (2019). Modelling of SAW-PDMS Acoustofluidics: Physical Fields and Particle Motions Influenced by Different Descriptions of the PDMS Domain. Lab Chip.

[9] Nama N., Barnkob R., Mao Z., Kähler C.J., Costanzo F., Huang T.J. (2015). Numerical Study of Acoustophoretic Motion of Particles in a PDMS Microchannel Driven by Surface Acoustic Waves. Lab Chip.

[10] Taatizadeh E., Dalili A., Rellstab-Sánchez P.I., Tahmooressi H., Ravishankara A., Tasnim N., Najjaran H., Li I.T., Hoorfar M. (2021). Micron-Sized Particle Separation with Standing Surface Acoustic Wave—Experimental and Numerical Approaches. Ultrason. Sonochem..

[11] Taatizadeh E., Dalili A., Tahmooressi H., Tasnim N., Li I.T.S., Hoorfar M. (2022). Nano-Scale Particle Separation with Tilted Standing Surface Acoustic Wave: Experimental and Numerical Approaches. Part. Part. Syst. Charact..

[12] Wang Y., Zhang Q., Tao R., Chen D., Xie J., Torun H., Dodd L.E., Luo J., Fu C., Vernon J. (2021). A Rapid and Controllable Acoustothermal Microheater Using Thin Film Surface Acoustic Waves. Sens. Actuators A Phys..

[13] Wang Y., Wu H., Li J., Tan W., Hu L. (2025). Self-Heating and Acoustothermal Heating in Microfluidics of LiNbO_3_ Surface Acoustic Wave Devices with Varying IDT Parameters. Sens. Actuators A Phys..

[14] Bai C., Zhou W., Yu S., Zheng T., Wang C. (2022). A Surface Acoustic Wave-Assisted Micromixer with Active Temperature Control. Sens. Actuators A Phys..

[15] Tseng W.-K., Lin J.-L., Sung W.-C., Chen S.-H., Lee G.-B. (2006). Active Micro-Mixers Using Surface Acoustic Waves on Y-Cut 128° LiNbO_3_. J. Micromech. Microeng..

[16] Bahat A., Tur-Kaspa I., Gakamsky A., Giojalas L.C., Breitbart H., Eisenbach M. (2003). Thermotaxis of Mammalian Sperm Cells: A Potential Navigation Mechanism in the Female Genital Tract. Nat. Med..

[17] Xiao W., Yu M., Yuan Y., Liu X., Chen Y. (2022). Thermotaxis of Mammalian Sperm. Mol. Hum. Reprod..

[18] Li Y., Wei S., Zheng T. (2021). Measurement of the Thermal Effect of Standing Surface Acoustic Waves in Microchannel by Fluoresence Intensity. Micromachines.

[19] Ozcelik A. (2021). Surface Acoustic Wave Induced Heat Knockdown of Caenorhabditis Elegans. Hittite J. Sci. Eng..

[20] Sridhar N., Fajrial A.K., Doser R.L., Hoerndli F.J., Ding X. (2022). Surface Acoustic Wave Microfluidics for Repetitive and Reversible Temporary Immobilization of C. Elegans. Lab Chip.

[21] Bahat A., Eisenbach M. (2006). Sperm Thermotaxis. Mol. Cell. Endocrinol..

[22] Doostabadi M.R., Mangoli E., Marvast L.D., Dehghanpour F., Maleki B., Torkashvand H., Talebi A.R. (2022). Microfluidic Devices Employing Chemo- and Thermotaxis for Sperm Selection Can Improve Sperm Parameters and Function in Patients with High DNA Fragmentation. Andrologia.

[23] Sarbaland F.b.N., Kobayashi M., Tanaka D., Fujita R., Tanaka N., Furuya M. (2025). Temperature Control in Microfluidic Devices: Approaches, Challenges, and Future Directions. Appl. Sci..

[24] Lei Y., Hu H. (2020). SAW-Driven Droplet Jetting Technology in Microfluidic: A Review. Biomicrofluidics.

[25] Zheng T., Wang C., Hu Q., Wei S. (2018). The Role of Electric Field in Microfluidic Heating Induced by Standing Surface Acoustic Waves. Appl. Phys. Lett..

[26] Huang Q.-Y., Sun Q., Hu H., Han J.-L., Lei Y.-L. (2021). Thermal Effect in the Process of Surface Acoustic Wave Atomization. Exp. Therm. Fluid Sci..

[27] Ha B.H., Lee K.S., Destgeer G., Park J., Choung J.S., Jung J.H., Shin J.H., Sung H.J. (2015). Acoustothermal Heating of Polydimethylsiloxane Microfluidic System. Sci. Rep..

[28] Jo M.C., Guldiken R. (2014). Effects of Polydimethylsiloxane (PDMS) Microchannels on Surface Acoustic Wave-Based Microfluidic Devices. Microelectron. Eng..

[29] Lyford T.J., Millard P.J., Da Cunha M.P. Cell Lysis Using Surface Acoustic Wave Devices for Sensor Applications. Proceedings of the 2012 IEEE International Ultrasonics Symposium.

[30] Shilton R.J., Mattoli V., Travagliati M., Agostini M., Desii A., Beltram F., Cecchini M. (2015). Rapid and Controllable Digital Microfluidic Heating by Surface Acoustic Waves. Adv. Funct. Mater..

[31] Han J., Yang F., Hu H., Huang Q., Lei Y., Li M. (2022). Thermal Control Design and Packaging for Surface Acoustic Wave Devices in Acoustofluidics. IEEE Trans. Ultrason. Ferroelectr. Freq. Control.

[32] Li L., Wu E., Jia K., Yang K. (2021). Temperature Field Regulation of a Droplet Using an Acoustothermal Heater. Lab Chip.

[33] Das P.K., Snider A.D., Bhethanabotla V.R. (2019). Acoustothermal Heating in Surface Acoustic Wave Driven Microchannel Flow. Phys. Fluids.

[34] Guo J., Kang Y., Ai Y. (2015). Radiation Dominated Acoustophoresis Driven by Surface Acoustic Waves. J. Colloid Interface Sci..

[35] Müller G., Möser M. (2013). Handbook of Engineering Acoustics.

[36] Frei W. (2017). Modeling Natural and Forced Convection in COMSOL Multiphysics. COMSOL Blog. https://www.comsol.com/blogs/modeling-natural-and-forced-convection-in-comsol-multiphysics.

[37] Gai J., Dervisevic E., Devendran C., Cadarso V.J., O’BRyan M.K., Nosrati R., Neild A. (2022). High-Frequency Ultrasound Boosts Bull and Human Sperm Motility. Adv. Sci..

[38] Chen S., Chen J., Qin Z., Wang J., Wang Y., Liu R., Zhao W., Zhang M., Zhang Y., Luo M. (2024). Microfluidic Thermotaxic Selection of Highly Motile Sperm and in Vitro Fertilization. Bio-Des. Manuf..

